# A GWAS-identified U-box E3 ligase MsPUB6 is positively regulated by MsDREB1C and contributes to drought tolerance in alfalfa

**DOI:** 10.1093/plphys/kiag679

**Published:** 2026-09-11

**Authors:** Yangyang Zhang, Xueqian Jiang, Huajuan Leng, Tian Zhang, Jing Cui, Ying Dong, Qingchuan Yang, Tiejun Zhang, Junmei Kang

**Affiliations:** Institute of Animal Science, Chinese Academy of Agricultural Sciences, No. 2 Yuanmingyuan West Road, Haidian District, Beijing 100193, China; Institute of Animal Science, Chinese Academy of Agricultural Sciences, No. 2 Yuanmingyuan West Road, Haidian District, Beijing 100193, China; Institute of Animal Science, Chinese Academy of Agricultural Sciences, No. 2 Yuanmingyuan West Road, Haidian District, Beijing 100193, China; Institute of Animal Science, Chinese Academy of Agricultural Sciences, No. 2 Yuanmingyuan West Road, Haidian District, Beijing 100193, China; Institute of Animal Science, Chinese Academy of Agricultural Sciences, No. 2 Yuanmingyuan West Road, Haidian District, Beijing 100193, China; Institute of Animal Science, Chinese Academy of Agricultural Sciences, No. 2 Yuanmingyuan West Road, Haidian District, Beijing 100193, China; Institute of Animal Science, Chinese Academy of Agricultural Sciences, No. 2 Yuanmingyuan West Road, Haidian District, Beijing 100193, China; Institute of Animal Science, Chinese Academy of Agricultural Sciences, No. 2 Yuanmingyuan West Road, Haidian District, Beijing 100193, China; Institute of Animal Science, Chinese Academy of Agricultural Sciences, No. 2 Yuanmingyuan West Road, Haidian District, Beijing 100193, China

## Abstract

U-box-type E3 ubiquitin ligases (PUBs) are critical for signaling homeostasis and abiotic stress responses via substrate ubiquitination, yet their upstream transcriptional regulation remains largely unexplored, especially in alfalfa drought adaptation. Through a genome-wide association study (GWAS) of 171 alfalfa accessions, we identified a novel U-box gene, *MsPUB6*, on chromosome 3, which encodes a protein with E3 ubiquitin ligase activity and is induced by drought, cold, salt, and abscisic acid. Overexpression and virus-induced gene silencing (VIGS) revealed that *MsPUB6* positively regulates drought tolerance by enhancing antioxidant enzymes (SOD and CAT), reducing malondialdehyde (MDA) and reactive oxygen species (ROS) levels, and maintaining chlorophyll content and leaf water content. Further mechanistic investigation showed that the transcription factor MsDREB1C specifically binds to the CRT/DRE element in the *MsPUB6* promoter and activates its transcription. CRISPR-Cas9-mediated *MsDREB1C* mutants exhibited a drought-sensitive phenotype similar to that of *MsPUB6*-silenced lines, accompanied by significantly reduced *MsPUB6* expression. In contrast, *MsDREB1C*-overexpressing lines showed enhanced drought tolerance and upregulated *MsPUB6* expression and similarly promoted drought tolerance by alleviating oxidative damage and membrane damage and by maintaining chlorophyll content and relative leaf water content. Collectively, this study reveals a novel regulatory module, MsDREB1C–*MsPUB6*, in which a DREB1 transcription factor positively regulates a U-box-type E3 ligase gene to contribute to drought tolerance in alfalfa. These findings not only provide important genetic resources for molecular breeding of drought-tolerant alfalfa but also expand our understanding of the functional diversity of plant U-box proteins.

## Introduction

Drought is one of the major abiotic stress factors limiting crop production worldwide, posing a serious threat to food security and agricultural sustainability (Gupta et al. 2020; Han et al. 2024). Alfalfa (*Medicago sativa* L.), a high-quality leguminous forage widely cultivated in arid and semi-arid regions of China, plays an indispensable role in livestock production and ecological restoration (Shi et al. 2024). However, this crop is highly sensitive to drought stress, and water deficit during the vegetative stage leads to significant reductions in yield and quality, severely constraining the sustainable development of the alfalfa industry (Yang et al. 2025). Therefore, identifying key drought-tolerance genes and elucidating their molecular mechanisms is a fundamental prerequisite for breeding novel drought-tolerant alfalfa varieties.

In response to drought stress, plants have evolved multilayered adaptive strategies encompassing morphological remodeling, physiological regulation, and molecular coordination (Gupta et al. 2020). These complex adaptive networks are coordinately regulated by key transcription factors, among which the AREB/ABF, NAC, WRKY, DOF, and DREB2 families act as core regulators that integrate upstream signals and activate downstream gene expression networks to precisely modulate drought tolerance (Chen et al. 2017; Mao et al. 2022; Kim et al. 2024; Ding et al. 2025). As core members of the DREB family, DREB1/CBF proteins play critical roles in plant abiotic stress responses by specifically recognizing CRT/DRE *cis*-acting elements and activating the expression of downstream stress-responsive genes (Kidokoro et al. 2021; Zhang and Xia 2023). Although DREB1/CBFs were initially identified as master regulators of cold signaling pathways (Kidokoro et al. 2022; Song et al. 2023), recent studies have revealed their involvement in drought adaptation in species such as soybean (*Glycine max*) and banana (*Musa acuminata*) (Zhou et al. 2020; Xu et al. 2022), suggesting that they may function as integrative hubs of multiple stress signaling networks. However, current studies on the drought tolerance function of DREB1/CBF have largely focused on overexpression-based validation, while the underlying fine-tuned transcriptional regulatory mechanisms remain unclear.

Plant U-box (PUB) proteins are important members of the E3 ubiquitin ligase family and are widely involved in the maintenance of signaling homeostasis and the regulation of stress responses (Trenner et al. 2022; Mou et al. 2024; Feng et al. 2026). Under drought stress, multiple PUB family members have been reported to participate in stress responses. For instance, loss-of-function mutants of *AtPUB46* and *AtPUB48* in *Arabidopsis thaliana* exhibit a hypersensitive phenotype to drought stress (Adler et al. 2017; Adler et al. 2018); TaPUB4 in wheat (*Triticum aestivum*) is involved in drought response through the abscisic acid (ABA) signaling pathway (Kim et al. 2023); heterologous overexpression of potato (*Solanum tuberosum*) *StPUB25* in Arabidopsis significantly enhances plant drought tolerance (Liu et al. 2024). In addition, several PUB proteins have been reported to function as negative regulators: overexpression of *AtPUB22* and *AtPUB23* in Arabidopsis leads to drought sensitivity, whereas loss-of-function mutants exhibit enhanced drought tolerance (Cho et al. 2008); heterologous expression of soybean *GmPUB6* in Arabidopsis negatively regulates drought response by suppressing ABA-mediated stomatal closure (Wang et al. 2020); and potato *StPUB27* promotes leaf water loss by positively regulating stomatal conductance (Tang et al. 2020). Beyond drought stress, PUB proteins also play important roles in cold stress, growth and development, and nodulation homeostasis (Mbengue et al. 2010; Liu et al. 2018; Chen et al. 2023; Wang et al. 2023a). Although these studies have revealed important functions of PUB proteins in stress responses across various plant species, the biological function and regulatory mechanisms of PUB family members in alfalfa remain unexplored.

In this study, a novel PUB family member, MsPUB6, was identified through a genome-wide association study (GWAS) of drought tolerance phenotypes in alfalfa. Overexpression and virus-induced gene silencing (VIGS) assays confirmed that *MsPUB6* positively regulates drought tolerance in alfalfa. Through further mechanistic exploration, the transcription factor MsDREB1C was found to specifically bind to the *MsPUB6* promoter and activate its transcription. Functional analyses further demonstrated that the MsDREB1C-*MsPUB6* module positively regulates drought tolerance in alfalfa by mitigating oxidative damage and membrane damage, maintaining chlorophyll content, and enhancing leaf water retention. Collectively, this study uncovers a novel mechanism by which a *PUB* gene is finely regulated at the transcriptional level, providing important insights into the multilayered regulatory network of U-box proteins in drought stress responses and offers a theoretical basis and key targets for the molecular breeding of drought-tolerant alfalfa.

## Results

### Identification and characterization of *MsPUB6*

Through GWAS, only one significantly associated marker (chr3_84606544, *P* = 3.9e-07) was identified on chromosome 3, explaining 15.68% of the phenotypic variation (Fig. 1a). This marker is located within a linkage disequilibrium (LD) block, which contains the gene *Msa.H.0162500* (Fig. 1a). Protein sequence alignment revealed that it shares 98.04% similarity with MtPUB6 from *Medicago truncatula*; it was therefore designated as MsPUB6 (Figure S1). The GWAS results were well calibrated, as indicated by the Q–Q plot (Fig. 1b). Haplotype analysis showed that the homozygous T/T haplotype exhibited significantly lower DRV_SLR values than the heterozygous T/A haplotype (t-test, *P* = 7.3e-07; Fig. 1c).

**Figure 1:**
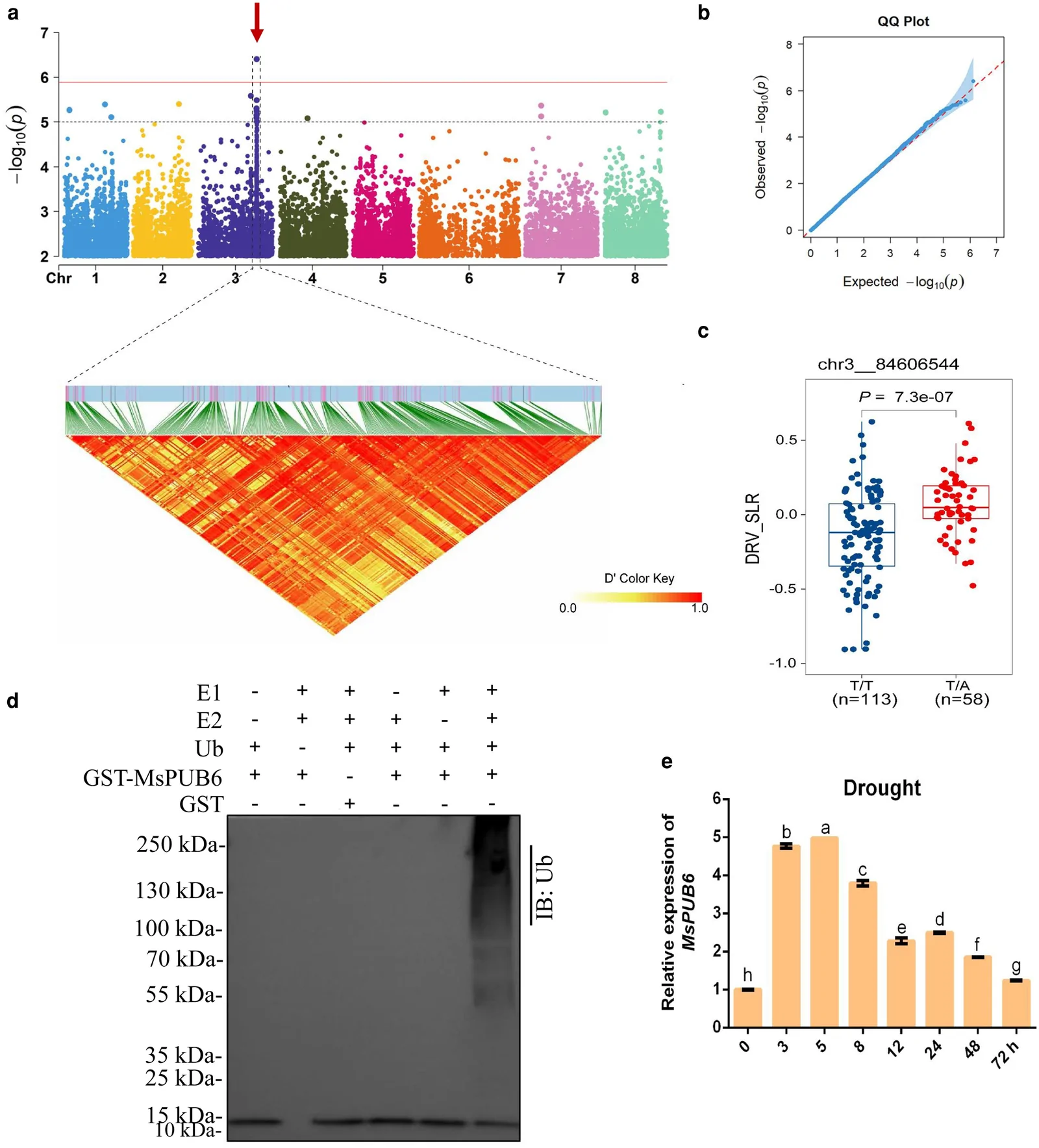
GWAS identifies *MsPUB6* as a candidate gene associated with drought tolerance in alfalfa, and its expression is induced by multiple abiotic stresses. a) Manhattan plot (upper panel) showing the genome-wide association signals for drought tolerance across 171 alfalfa accessions. A significant peak exceeding the threshold is observed on chromosome 3, corresponding to the marker chr3_84606544. The lower panel displays the local linkage disequilibrium (LD) heatmap around this peak, with the red block indicating the LD region containing the candidate gene *Msa.H.0162500* (*MsPUB6*). b) Q–Q plot of the observed versus expected -log_10_ (*P*) values. c) Haplotype analysis of the significant SNP (chr3_84606544). d) In vitro ubiquitination assay of GST-MsPUB6 (251 to 550 aa). e) RT-qPCR analysis of *MsPUB6* expression under drought treatments. Three-week-old hydroponically grown alfalfa seedlings were subjected to15% PEG6000. Error bars represent the mean ± standard error (SEM) of 3 biological replicates. Statistical significance among groups was assessed by one-way ANOVA, and different lowercase letters indicate significant differences (*P* < 0.05) based on Duncan's multiple range test.

Given that MsPUB6 possesses a conserved U-box domain (Figure S1) and belongs to the E3 ubiquitin ligase family, we sought to determine whether MsPUB6 also exhibits ubiquitin ligase activity. An in vitro ubiquitination system was employed to evaluate the E3 ubiquitin ligase catalytic activity of the truncated MsPUB6 (251 to 550 aa) protein. The reaction mixtures were assembled with the following components: E1 ubiquitin-activating enzyme, E2 ubiquitin-conjugating enzyme, ubiquitin (Ub), recombinant GST-MsPUB6 (251 to 550 aa) protein, and GST protein from the empty vector as a negative control (Fig. 1d). The results demonstrated that a high-molecular-weight smear of polyubiquitinated products was detected by an anti-Ub antibody only in the complete reaction containing all essential components (E1, E2, Ub, and GST-MsPUB6). In contrast, no obvious polyubiquitin chain signals were observed in the reactions lacking any one of E1, E2, or Ub, nor in the control group supplemented with GST protein alone. These findings indicate that the GST-MsPUB6 recombinant protein catalyzes the formation of polyubiquitin chains in a manner strictly dependent on the complete ubiquitination system, thereby confirming that MsPUB6 possesses typical E3 ubiquitin ligase catalytic activity.

Having established its biochemical function, we next investigated its expression patterns to gain insights into its potential physiological roles. Tissue-specific expression analysis revealed that *MsPUB6* was expressed in roots, stems, and leaves, with the highest expression level in stems, followed by leaves, and the lowest in roots (Figure S2). Furthermore, expression pattern analysis showed that *MsPUB6* was induced by drought, cold, salt, and ABA treatments, displaying a dynamic expression pattern that increased initially and then decreased over time (Fig. 1e and Figure S3). These results suggest that *MsPUB6* is broadly involved in abiotic stress responses and may be subject to fine-tuned regulation.

### Overexpression of *MsPUB6* enhances drought tolerance in alfalfa

To investigate the biological function of *MsPUB6*, we generated stable *MsPUB6*-overexpressing (*MsPUB6*-OE) lines via Agrobacterium-mediated transformation. Quantitative real-time PCR (qRT-PCR) analysis revealed that *MsPUB6* transcript levels in OE19 and OE20 were 4.1- and 2.6-fold higher than those in wild-type (WT), respectively (Figure S4a); both lines were selected for subsequent phenotypic analyses. Prior to drought treatment, uniformly grown WT and *MsPUB6*-OE cuttings (Fig. 2a) were transplanted into individual pots (8.5 × 8.5 × 9.5 cm) containing pre-weighed standard growth substrate (150 ± 5 g; vermiculite: nutrient soil = 2:1). All pots were fully saturated with water, and excess water was removed from the trays the following day. Drought stress was then imposed by withholding irrigation, and pots were rotated every 2 to 3 d to minimize positional effects. After 1 month of drought treatment, WT plants exhibited severe leaf wilting and yellowing, whereas *MsPUB6*-OE plants maintained greener leaves with better water retention (Fig. 2b). Following cutting and re-watering for 7 d, only approximately one-third of WT plants recovered, whereas all *MsPUB6*-OE plants survived (Fig. 2c), indicating that overexpression of *MsPUB6* significantly enhances drought tolerance in alfalfa.

**Figure 2:**
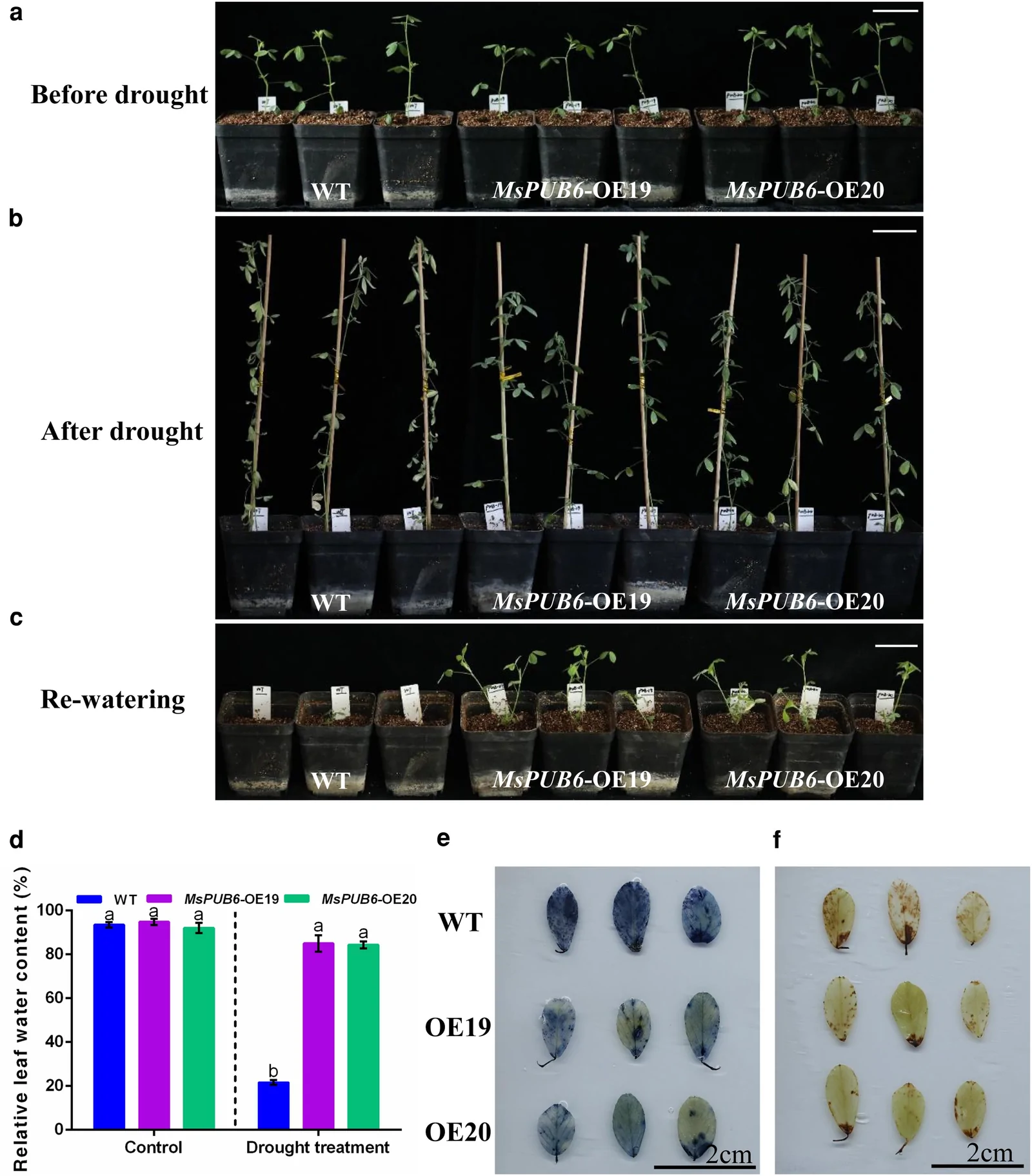
Overexpression of *MsPUB6* enhances drought tolerance in alfalfa. a to c) Phenotypic comparison of wild-type (WT) and *MsPUB6*-overexpressing (*MsPUB6*-OE) lines before treatment (a), after drought stress (b), and after cutting and re-watering (c). Uniformly grown cuttings were transplanted into plastic pots (8.5 × 8.5 × 9.5 cm) containing equal amounts of growth substrate (vermiculite: nutrient soil = 2:1), and the substrate was fully saturated with water before transplantation. After transplantation, plants were subjected to natural drought for 1 month, followed by cutting and then re-watering for 1 week. Scale bars = 5 cm. d) Comparison of relative leaf water content after drought treatment. e) NBT staining for detection of superoxide anion (O_2_^-^) accumulation in leaves after drought treatment. (f) DAB staining for detection of hydrogen peroxide (H_2_O_2_) accumulation in leaves after drought treatment. Error bars represent the mean ± standard error (SEM) of 3 biological replicates. Statistical significance among groups was assessed by one-way ANOVA, and different lowercase letters indicate significant differences (*P* < 0.05) based on Duncan's multiple range test.

Physiological analyses further supported this conclusion. Under drought stress, the relative leaf water content of WT plants dropped to 22%, while that of *MsPUB6*-OE plants remained at 84% to 85% (Fig. 2d). NBT and DAB staining revealed significantly reduced accumulation of superoxide anions (O_2_^-^) and hydrogen peroxide (H_2_O_2_) in *MsPUB6*-OE leaves compared with WT (Fig. 2e–f). Before drought treatment, no significant differences in physiological parameters were observed between WT and *MsPUB6*-OE plants. However, after drought stress, *MsPUB6*-OE lines exhibited significantly lower malondialdehyde (MDA) and H_2_O_2_ contents and significantly higher catalase (CAT) activity than WT (Figure S4). Collectively, these results suggest that overexpression of *MsPUB6* alleviates oxidative damage and membrane damage while enhancing leaf water retention, thereby contributing to improved drought tolerance in alfalfa.

### Knockdown of *MsPUB6* reduces drought tolerance in alfalfa

To further investigate the role of MsPUB6 in drought tolerance, we generated *MsPUB6*-silenced lines using virus-induced gene silencing. qRT-PCR analysis showed that *MsPUB6* transcript levels in TRV2-*MsPUB6* plants were reduced to 39% of those in the empty vector control (TRV2) (Fig. 3c), confirming effective silencing of the target gene. Subsequently, both control and silenced lines were subjected to drought stress. Phenotypic observations revealed that TRV2-*MsPUB6* plants exhibited more severe leaf wilting than the control, particularly with the pronounced shrinkage and abscission of basal leaves (Fig. 3a). Examination of the apical leaves further showed that *MsPUB6*-silenced plants displayed greater water loss and obvious leaf yellowing (Fig. 3b). These phenotypic differences suggested that silencing *MsPUB6* may compromise the plant's ability to withstand drought stress. To explore the underlying physiological mechanisms, we measured relevant physiological parameters before and after drought treatment. No significant difference in electrolyte leakage was observed between the control and silenced lines prior to drought stress. However, after drought treatment, conductivity in TRV2-*MsPUB6* plants increased to 79%, significantly higher than that in the control (35%), indicating exacerbated membrane damage upon *MsPUB6* silencing (Fig. 3d). In parallel, chlorophyll content was significantly lower in the silenced lines than in the control (Fig. 3e). Collectively, these results demonstrate that knockdown of *MsPUB6* reduces drought tolerance in alfalfa by aggravating membrane damage and accelerating chlorophyll degradation.

**Figure 3:**
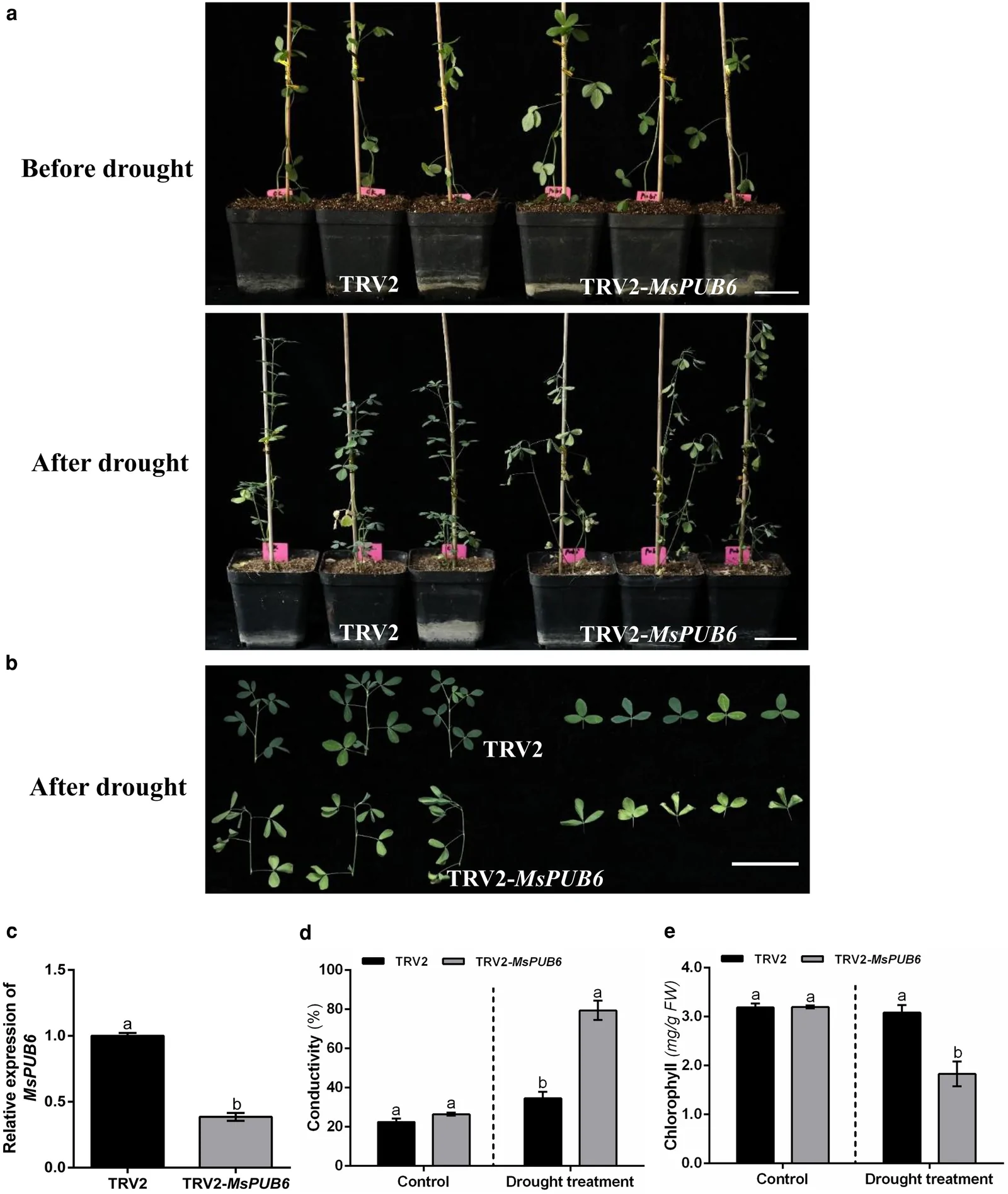
Knockdown of *MsPUB6* reduces drought tolerance in alfalfa. a) Phenotypic comparison of control (TRV2) and *MsPUB6*-silenced lines (TRV2-*MsPUB6*) under drought stress. Scale bars = 5 cm. b) Leaf morphology observation of control and silenced lines after drought stress. Scale bars = 5 cm. c) qRT-PCR analysis of *MsPUB6* silencing efficiency. d to e) Measurement of leaf conductivity (d) and chlorophyll content (e) in control and silenced lines after drought stress. Error bars represent the mean ± standard error (SEM) of 3 biological replicates. Statistical significance among groups was assessed by one-way ANOVA, and different lowercase letters indicate significant differences (*P* < 0.05) based on Duncan's multiple range test.

### MsDREB1C binds to and activates the transcription of *MsPUB6*

Having established the role of MsPUB6 in drought tolerance, we next investigated its upstream regulatory mechanism. Promoter element (Table S1) analysis suggested that the transcription factor MsDREB1C (Zhang et al. 2024) may act as a potential upstream regulator of *MsPUB6*. To test this hypothesis, we first performed Electrophoretic mobility shift assays (EMSA) and found that MsDREB1C specifically bound to the CRT/DRE element (GCCGAC) within the *MsPUB6* promoter. This binding was competitively reduced by unlabeled probes in a dose-dependent manner and was abolished when the core element was mutated (Fig. 4a), indicating that the MsDREB1C specifically recognizes the CRT/DRE element in the *MsPUB6* promoter.

**Figure 4:**
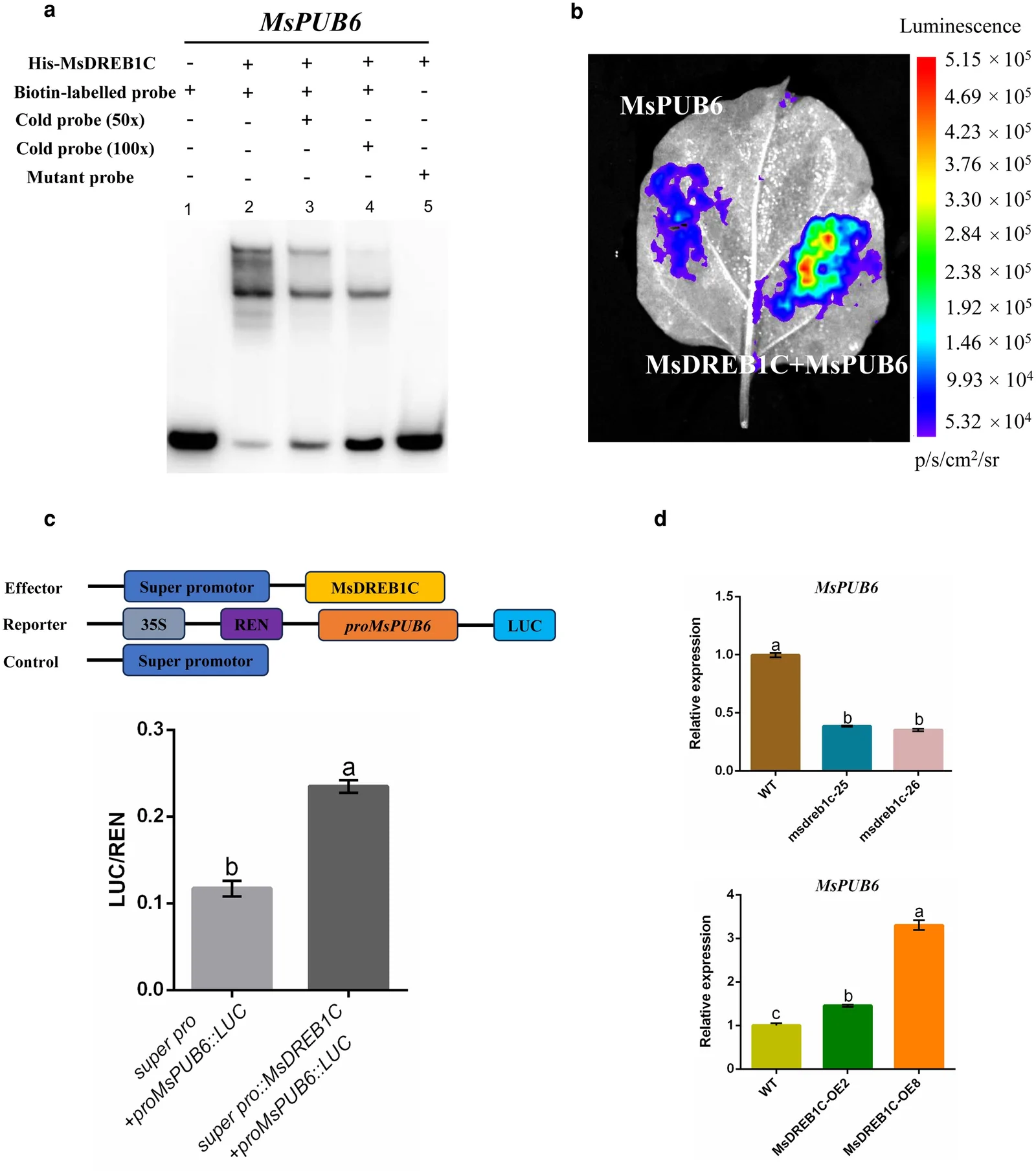
Transcription factor MsDREB1C specifically binds to and activates the expression of *MsPUB6*. a) EMSA analysis of the in vitro binding of MsDREB1C protein to the CRT/DRE element in the *MsPUB6* promoter. “+” and “–” indicate the presence or absence of the corresponding component in the reaction system, respectively. b) Imaging showing the activation effect of MsDREB1C on the *MsPUB6* promoter activity. c) Quantitative analysis of the regulatory effect of MsDREB1C on the transcriptional activity of the *MsPUB6* promoter using a dual-luciferase reporter system. d) qRT-PCR analysis of *MsPUB6* expression levels in wild-type (WT), *MsDREB1C*-overexpressing (*MsDREB1C*-OE), and gene-edited mutant (*msdreb1c*) lines. Error bars represent the mean ± standard error (SEM) of 3 biological replicates. Statistical significance among groups was assessed by one-way ANOVA, and different lowercase letters indicate significant differences (*P* < 0.05) based on Duncan's multiple range test.

To assess the transcriptional regulatory activity of MsDREB1C on *MsPUB6*, dual-luciferase reporter assays were performed. Luciferase (LUC) imaging revealed that MsDREB1C significantly activated *MsPUB6* promoter-driven luciferase expression (Fig. 4b). Quantitative analysis showed that co-expression of MsDREB1C resulted in a LUC/REN ratio that was approximately 2-fold that of the empty vector control, confirming that MsDREB1C significantly enhances the transcriptional activity of *MsPUB6* (Fig. 4c).

To validate this regulatory relationship at the genetic level, we generated *MsDREB1C* mutants (*msdreb1c*) using CRISPR-Cas9 and performed qRT-PCR analysis together with existing overexpression lines. *MsPUB6* transcript levels were significantly upregulated in *MsDREB1C*-overexpressing lines and significantly downregulated in *msdreb1c* mutants (Fig. 4d), further supporting that *MsPUB6* expression is positively regulated by MsDREB1C. Collectively, these results demonstrate that MsDREB1C binds to the promoter of *MsPUB6* and activates its transcription.

### MsDREB1C positively regulates drought tolerance in alfalfa

To investigate the biological function of the upstream transcription factor MsDREB1C, both *MsDREB1C*-edited mutants and overexpression lines were employed to evaluate the drought tolerance, respectively.

The targeting sites for the 2 gRNAs within the *MsDREB1C* gene, which is composed of a single exon and lacks introns, are indicated in Fig. 5a. Genomic DNA fragments flanking the target sites were amplified and subjected to colony sequencing. Two mutant lines, *msdreb1c*-25 and *msdreb1c*-26, were identified (Figure S5 and Table S2), with 4 and 6 distinct mutation types, respectively (Fig. 5a). Under the same drought treatment regime as described above, *msdreb1c* mutants exhibited significantly reduced drought tolerance compared with WT plants after 1 month of water deficit, manifesting as severe wilting, yellowing, and failure to recover upon re-watering (Fig. 5b). Physiological measurements further revealed that chlorophyll content and relative leaf water content were significantly lower in the mutants than in WT after drought stress (Fig. 5c–d), whereas malondialdehyde (MDA) and hydrogen peroxide (H_2_O_2_) levels were significantly higher, and superoxide dismutase (SOD) and catalase (CAT) activities were significantly lower (Figure S6). These results indicate that loss of *MsDREB1C* exacerbates oxidative damage and membrane damage under drought stress, thereby compromising drought tolerance in alfalfa.

**Figure 5:**
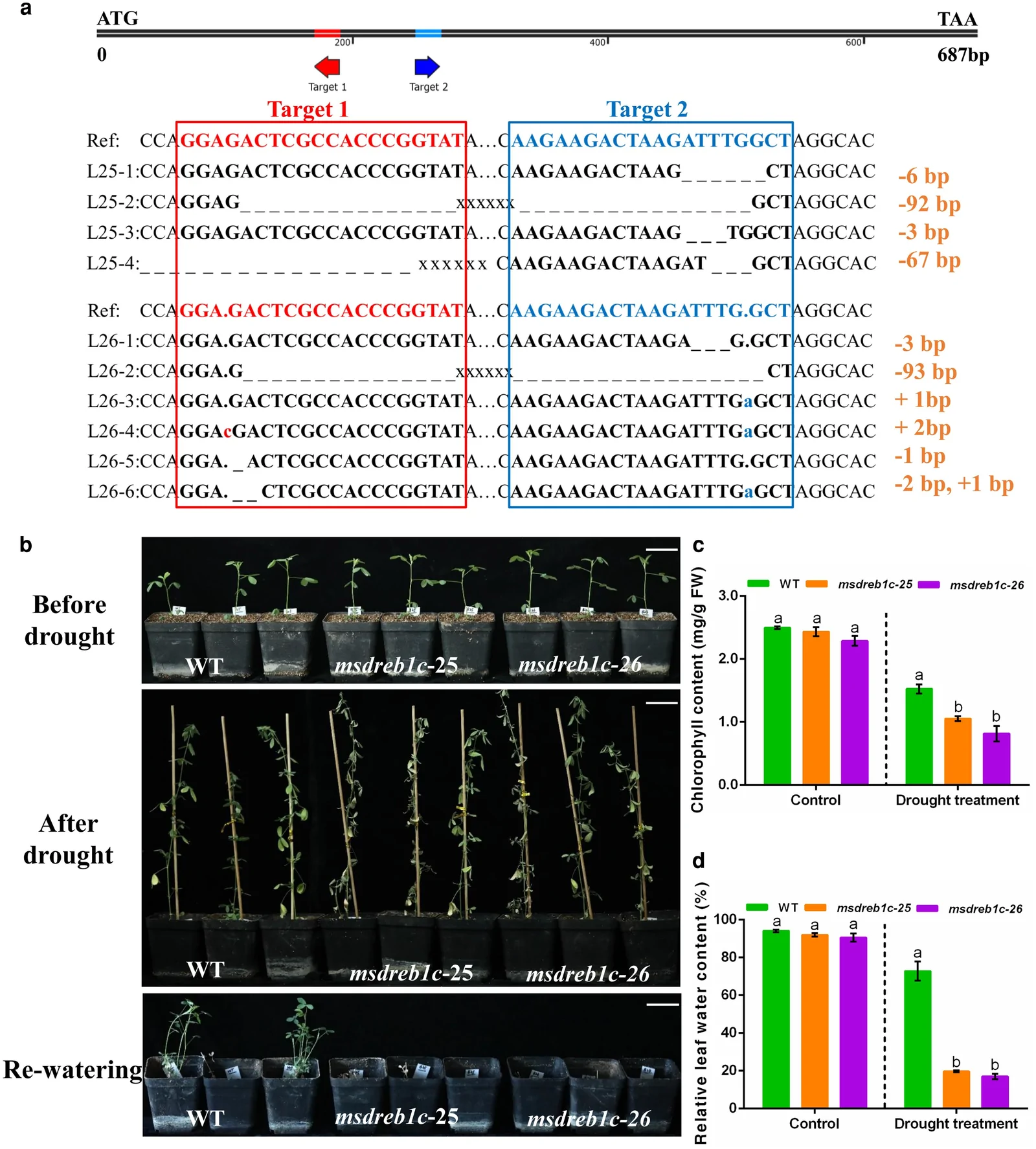
CRISPR-Cas9-mediated *msdreb1c* mutants exhibit enhanced susceptibility to drought stress in alfalfa. a) Gene structure of *MsDREB1C* and the locations of 2 editing targets (Target 1 and Target 2). The 2 gene-edited lines, *msdreb1c*-25 and *msdreb1c*-26, exhibited 4 and 6 distinct mutation types at Target 1 and Target 2, respectively (underscore “_” indicates nucleotide deletion, “xxxxx” indicates large fragment deletion, and lowercase letters indicate nucleotide insertion). b) Phenotypic comparison of wild-type (WT) and *msdreb1c* mutant lines under drought stress followed by cutting and subsequent re-watering. Scale bars = 5 cm. c) Measurement of leaf chlorophyll content after drought treatment. d) Measurement of leaf relative water content after drought treatment. Error bars represent the mean ± standard error (SEM) of 3 biological replicates. Statistical significance among groups was assessed by one-way ANOVA, and different lowercase letters indicate significant differences (*P* < 0.05) based on Duncan's multiple range test.

In addition, the drought tolerance of *MsDREB1C*-overexpressing lines was further evaluated. Previous studies have shown that MsDREB1C is involved in regulating growth and quality in alfalfa (Zhang et al. 2024). Under the same drought treatment regime as described above, 2 overexpression lines (OE2 and OE8) and WT plants were subjected to phenotypic analysis. The results showed that *MsDREB1C*-OE lines exhibited significantly enhanced drought tolerance compared with WT (Fig. 6a). Physiological measurements further revealed that, after drought stress, the overexpression lines displayed reduced membrane damage and oxidative stress levels, along with enhanced leaf water retention and increased antioxidant enzyme activities, relative to WT (Fig. 6b–g). Collectively, these results demonstrate that overexpression of *MsDREB1C* significantly enhances drought tolerance in alfalfa.

**Figure 6:**
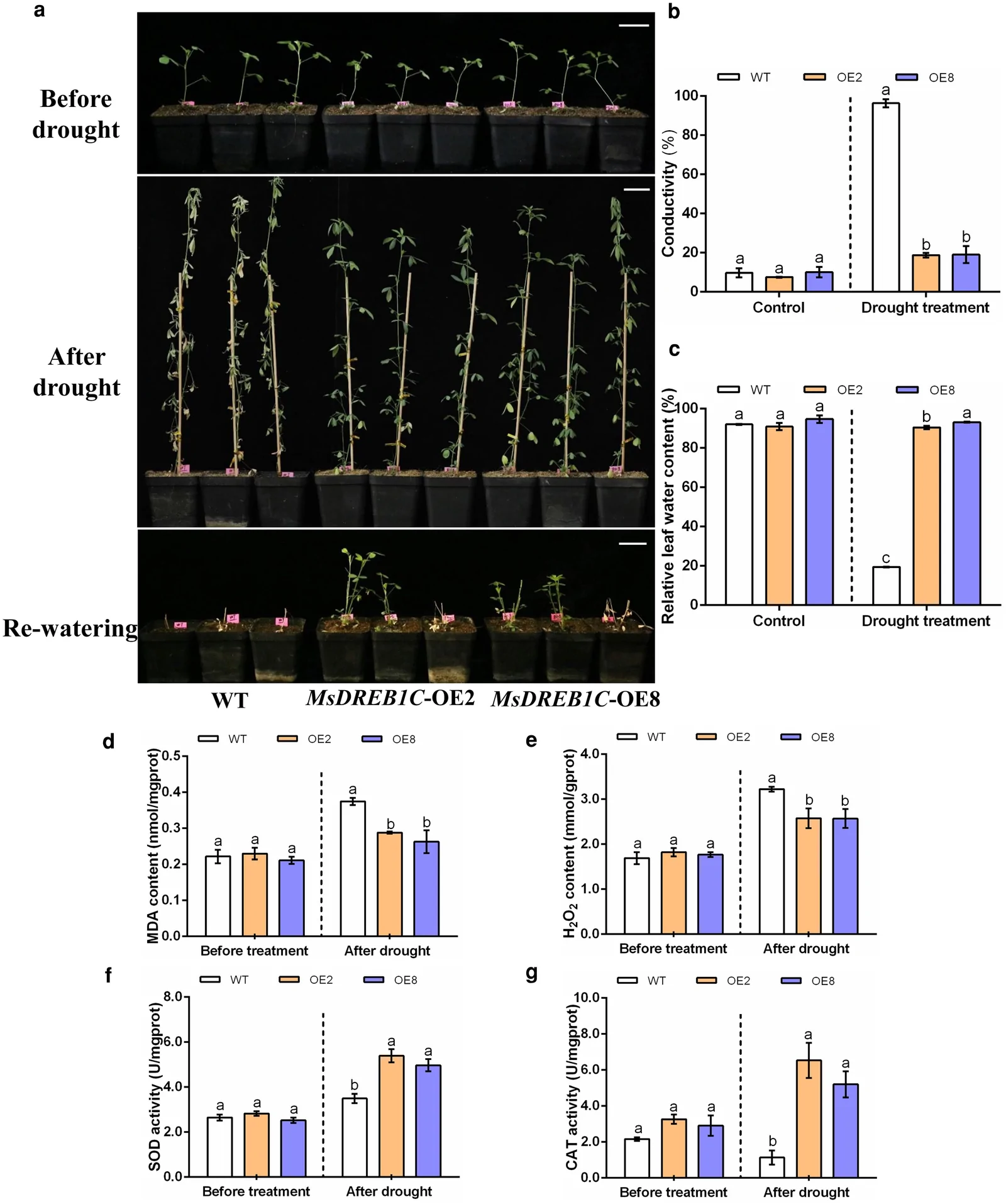
Overexpression of *MsDREB1C* enhances drought tolerance in alfalfa. a) Phenotypic comparison of wild-type (WT) and *MsDREB1C*-overexpressing (*MsDREB1C*-OE) lines under drought stress followed by cutting and subsequent re-watering. Scale bars = 5 cm. b) Measurement of leaf electrolyte leakage after drought treatment. c) Measurement of leaf relative water content after drought treatment. d) Measurement of malondialdehyde (MDA) content after drought treatment. e) Measurement of hydrogen peroxide (H_2_O_2_) content after drought treatment. f) Measurement of superoxide dismutase (SOD) activity after drought treatment. g) Measurement of catalase (CAT) activity after drought treatment. Error bars represent the mean ± standard error (SEM) of 3 biological replicates. Statistical significance among groups was assessed by one-way ANOVA, and different lowercase letters indicate significant differences (*P* < 0.05) based on Duncan's multiple range test.

Taken together, these findings establish MsDREB1C as a positive regulator that plays a critical role in drought tolerance in alfalfa.

## Discussion

### Functional diversity of U-box proteins in drought tolerance regulation

U-box-type E3 ubiquitin ligases exhibit functional diversity in plant drought responses, functioning as either positive or negative regulators. In Arabidopsis, *AtPUB22* and *AtPUB23* act as negative regulators; their overexpression leads to drought sensitivity, whereas loss-of-function mutants display enhanced drought tolerance (Cho et al. 2008). In contrast, *AtPUB46* and *AtPUB48* function as positive regulators, with their T-DNA insertion mutants showing a hypersensitive phenotype under drought stress (Adler et al. 2017; Adler et al. 2018). Similar functional divergence is observed in potato: *StPUB27* acts as a negative regulator by positively regulating stomatal conductance to promote leaf water loss, and its downregulation significantly enhances plant drought tolerance (Tang et al. 2020); whereas heterologous overexpression of *StPUB25* in Arabidopsis significantly improves plant drought tolerance (Liu et al. 2024), indicating its role as a positive regulator. In this study, through GWAS, we identified a novel U-box-type E3 ubiquitin ligase gene, *MsPUB6*, in alfalfa. Overexpression of *MsPUB6* significantly enhanced drought tolerance, whereas VIGS silencing reduced drought tolerance (Figs. 2 and 3). This result is consistent with the functions of positive regulators such as *AtPUB46* and *StPUB25*, further supporting the functional diversity of U-box proteins in drought tolerance regulation. Notably, this functional divergence may arise from differences in substrate specificity or the signaling pathways in which different PUB proteins operate, suggesting that U-box proteins have evolved diverse regulatory strategies adapted to different drought response requirements.

### MsPUB6 enhances drought tolerance by alleviating oxidative damage and membrane damage

Under drought stress, reactive oxygen species (ROS) exhibit a dual biological effect. Moderate ROS production serves as a key signaling molecule in stress responses, for example, by promoting stomatal closure to enhance drought tolerance (Gao et al. 2022; Yang et al. 2024). However, excessive ROS accumulation triggers oxidative damage and membrane lipid peroxidation, which are major mechanisms leading to cellular injury (Wei et al. 2019; Li et al. 2023). In this study, we found that alfalfa overexpressing *MsPUB6* exhibited significantly reduced levels of MDA, O_2_^-^, and H_2_O_2_; increased antioxidant enzyme activity; higher relative leaf water content; and maintained chlorophyll content after drought stress (Fig. 2 and Figure S4). These results indicate that MsPUB6 enhances drought tolerance by boosting antioxidant enzyme activity, alleviating membrane damage, and maintaining water balance. Similarly, rice (*Oryza sativa*) *OsPUB15* enhances salt tolerance by reducing ROS levels and oxidative stress to maintain cell survival (Park et al. 2011); Arabidopsis *AtPUB2* positively regulates oxidative stress tolerance by enhancing antioxidant enzyme activity and reducing ROS and MDA accumulation (Saini et al. 2023). Taken together, alleviating oxidative damage by strengthening antioxidant defense may be a conserved mechanism through which PUB proteins positively regulate stress tolerance. Nevertheless, whether MsPUB6 directly modulates antioxidant enzyme activity or acts indirectly through ubiquitin-mediated degradation of certain negative regulators remains to be further investigated.

### A novel transcriptional regulatory mode in which *MsPUB6* is positively regulated by MsDREB1C

Compared with the extensive studies on the DREB2 family in drought responses (Liu et al. 1998; Kim et al. 2024; Dai et al. 2026), the DREB1/CBF family is primarily recognized as a master regulator of cold stress (Kidokoro et al. 2022; Wang et al. 2023b), and direct functional evidence for its involvement in drought stress remains relatively limited, especially in the leguminous forage alfalfa. In this study, we obtained *MsDREB1C* gene-edited mutants (*msdreb1c*-25 and *msdreb1c*-26) using CRISPR-Cas9 technology. Phenotypic evaluation under drought stress revealed that the mutants exhibited significant sensitivity to drought, manifesting as plant wilting, a significantly lower survival rate after re-watering compared with the wild-type, as well as decreased chlorophyll content and relative water content, and increased MDA and H_2_O_2_ levels (Fig. 5 and Figure S6). This phenotype was highly consistent with the drought-sensitive phenotype of *MsPUB6*-silenced lines. Together with the significantly enhanced drought tolerance observed in *MsDREB1C*-overexpressing lines, these genetic evidences strongly indicate that MsDREB1C is a positive regulator of drought tolerance in alfalfa.

Previous studies on U-box-type E3 ubiquitin ligases have largely focused on how they degrade substrate proteins as E3 ligases, thereby participating in growth and development, signal transduction, and stress responses. For example, Arabidopsis *PUB30* and *PUB31* precisely regulate inflorescence development and stomatal differentiation by mediating the ubiquitin-dependent degradation of the ERECTA receptor kinase (Chen et al. 2023); wheat (*T. aestivum*) *TaPUB1* negatively regulates ABA responses and seed development by targeted degradation of key ABA signaling components TaPYL4 and TaABI5 (Zhang et al. 2021); and *M. truncatula* MtPUB1 and MtPUB2 maintains nodule number homeostasis by interacting with symbiotic receptor kinases (Mbengue et al. 2010; Liu et al. 2018). However, the transcriptional regulation of *PUB* genes themselves, ie, how upstream transcription factors regulate *PUB* gene expression, has long been underexplored.

In this study, we found that the promoter of *MsPUB6* contains a CRT/DRE *cis*-acting element that is specifically bound and activated by the transcription factor MsDREB1C (Fig. 4). This mode of a DREB1-type transcription factor positively regulating an E3 ligase gene has rarely been reported. Notably, a recent study revealed another direction of regulation between PUB proteins and transcription factors: Arabidopsis AtPUB25 and AtPUB26 catalyze 2 distinct types of ubiquitin chains, K48 and K63, to differentially modify the transcription factors ICE1 and MYB15, thereby dynamically controlling the timing and intensity of CBF expression under cold stress (Wang et al. 2023a). Thus, in plant responses to abiotic stress, PUB proteins not only serve as downstream effectors regulated by transcription factors but also feedback-regulate upstream transcription factors through posttranslational modifications.

Combining our findings with the above discoveries outlines a bidirectional regulatory mode. On one hand, transcription factors (eg, MsDREB1C) directly activate the transcription of *PUB* genes, achieving rapid mobilization of E3 ligases at the transcriptional level. On the other hand, PUB proteins (eg, AtPUB25/26) fine-tune signal strength through ubiquitin-mediated modification of transcription factors (Wang et al. 2023a). Such bidirectional interplay may represent a common and efficient regulatory strategy in plant stress signaling networks.

Our study provides direct genetic evidence supporting the involvement of the DREB1 family in drought responses and reveals a previously unrecognized mechanism in alfalfa, in which a DREB1-type transcription factor regulates drought tolerance by activating a U-box-type E3 ligase gene. Nevertheless, we acknowledge that MsDREB1C likely regulates multiple downstream targets beyond *MsPUB6*, with *MsPUB6* being one of the several responsive factors rather than the sole executor of drought tolerance. The relatively moderate transcriptional induction of *MsPUB6* in MsDREB1C-OE lines (Fig. 4d) suggests a fine-tuning rather than a switch-like activation mode, consistent with the presence of multiple stress-related *cis*-elements in the *MsPUB6* promoter (Table S1) that may integrate inputs from other signaling pathways. Furthermore, given that MsPUB6 itself possesses E3 ubiquitin ligase activity (Fig. 1d), the identification of ubiquitination substrates of MsPUB6 will be essential to fully elucidate its molecular regulatory network in drought responses. In addition, the relationship between natural variation at the chr3_84606544 locus and *MsPUB6* transcript abundance across diverse germplasm represents an important direction for future studies.

## Conclusion

In summary, this study identified a novel drought tolerance candidate gene, *MsPUB6*, in alfalfa through GWAS, and revealed the positive regulatory role of the transcriptional module MsDREB1C–*MsPUB6* in drought tolerance. This module enhances drought tolerance by alleviating oxidative damage and membrane damage, maintaining chlorophyll content, and improving leaf water retention capacity (Fig. 7). By establishing a direct link between a transcription factor and a U-box-type E3 ligase, these findings uncover the upstream transcriptional regulatory mechanism of U-box proteins. This work not only broadens our understanding of the functional diversity of plant U-box proteins but also provides key molecular targets and a theoretical basis for molecular breeding of drought-tolerant alfalfa.

**Figure 7:**
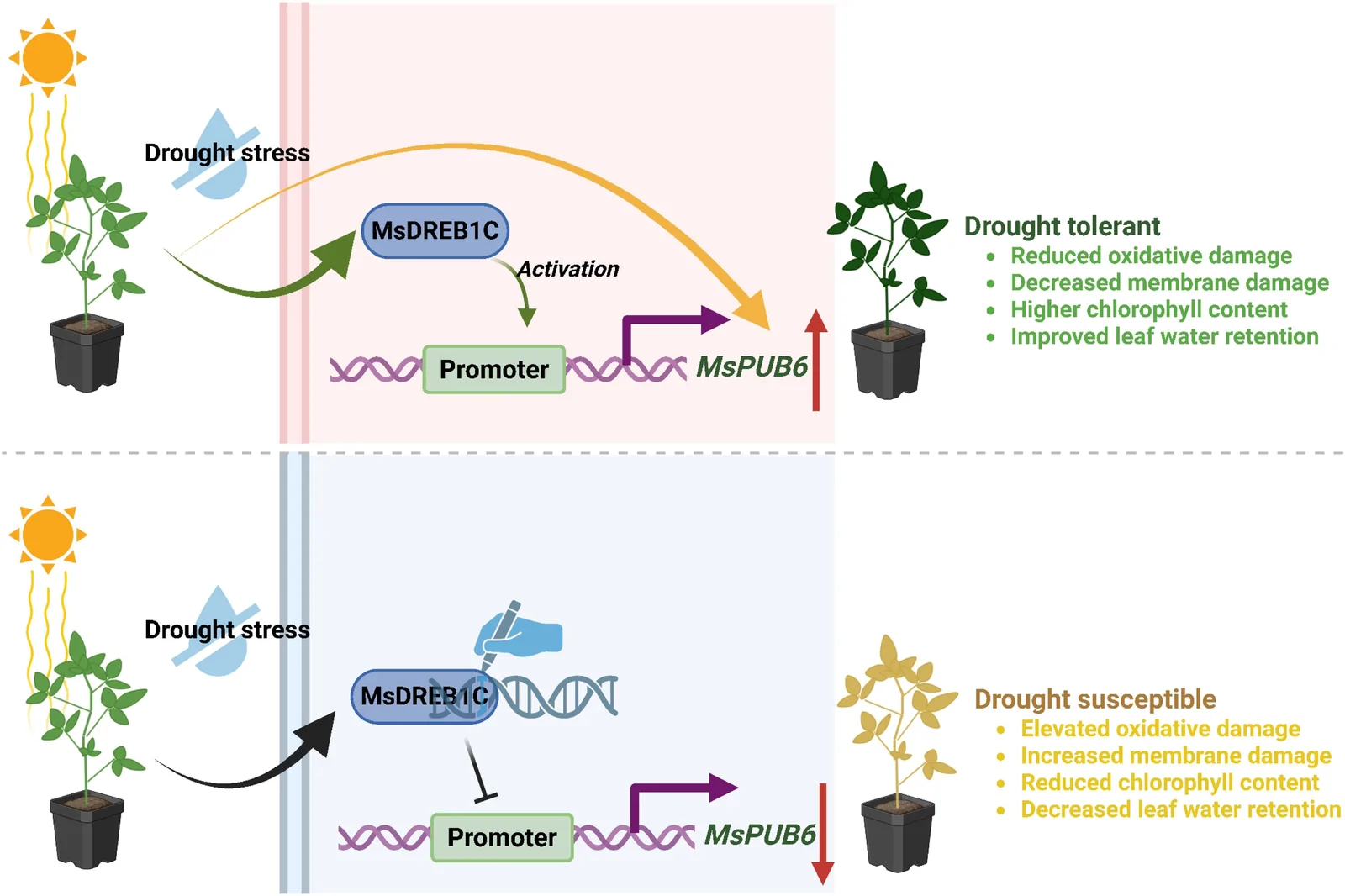
Schematic diagram illustrating the molecular mechanism by which the MsDREB1C-*MsPUB6* module regulates drought tolerance in alfalfa. Under drought stress, the expression of the transcription factor MsDREB1C is induced, which binds to the CRT/DRE element in the promoter of its downstream target gene *MsPUB6*, activating its transcription. *MsPUB6* enhances drought tolerance by mitigating oxidative damage and membrane damage, maintaining chlorophyll content, and improving leaf water retention. Conversely, in *MsDREB1C*-edited mutants, this regulatory pathway is disrupted, leading to reduced *MsPUB6* expression, increased oxidative and membrane damage, decreased chlorophyll content, and lower leaf water content, ultimately resulting in a drought-sensitive phenotype. The schematic diagram was created in BioRender. Zhang, Y. (2026) https://BioRender.com/0nvxy2l under an academic license.

## Materials and methods

### GWAS analysis for drought response

The drought response value (DRV) of the stem-to-leaf ratio (DRV_SLR) was used as the phenotypic trait for GWAS. The stem-to-leaf ratio (SLR) reflects above-ground morphological plasticity under water deficit, as plants typically suppress leaf expansion and maintain stem growth in response to drought stress (Gupta et al. 2020).

The association panel, growth conditions, and single nucleotide polymorphism (SNP) calling followed the procedures described in our previous study (Jiang et al. 2025). Briefly, the SLR of the association panel was measured under both control and drought conditions in a controlled greenhouse environment, and calculated the drought response value (DRV) for GWAS.

The drought response value (DRV) for SLR was calculated using the following formula:


DRV_SLR=(SLR_control–SLR_drought)/SLR_control

GWAS was performed for the DRV_SLR trait using TASSEL software with a generalized linear model (GLM), based on 1,303,374 high-quality SNPs (Bradbury et al. 2007). The suggestive threshold was set at 5.89, as determined by the Genetic Type I Error Calculator (GEC; http://pmglab.top/gec/#/).

### Plant materials and growth conditions

Alfalfa (*M. sativa* L. ′Zhongmu No.1') was grown under controlled environmental conditions, including a 16 h light/8 h dark photoperiod, a day/night temperature regime of 22 °C/20 °C (actual temperatures varied within a range of ± 2 to 3 °C depending on ambient conditions), a light intensity of 65 µmol m^−2^ s^−1^, and 50% to 60% relative humidity. Plants were maintained in soil culture within a greenhouse or in hydroponic culture within a growth chamber, depending on the experimental requirements.

### Abiotic stress treatments and expression analysis

Alfalfa seeds with uniform size and plumpness were surface-sterilized and germinated on moist filter paper in Petri dishes. After 7 d of growth under controlled conditions, seedlings of consistent size were transferred to hydroponic culture with 1/2 Hoagland nutrient solution (pH 5.95). For tissue-specific expression analysis, roots, stems, and leaves were collected from 3-week-old plants. For stress treatments, 3-week-old seedlings were subjected to cold (4 °C), drought (15% PEG6000), salt (200 mM NaCl), or ABA (100 μM) treatments. Leaf samples were collected at 0, 3, 5, 8, 12, 24, 48, and 72 h posttreatment, immediately frozen in liquid nitrogen, and stored for subsequent gene expression analysis.

### Total RNA isolation, reverse transcription, and qRT-PCR analysis

Total RNA was extracted from alfalfa tissues using the Eastep Total RNA Extraction Kit (Promega, United States) according to the manufacturer's instructions. RNA concentration and purity were assessed using a NanoDrop spectrophotometer (Thermo Fisher Scientific, United States), and RNA integrity was verified by 1% agarose gel electrophoresis. First-strand cDNA was synthesized from 1 µg of total RNA with the HiScript III All-in-one RT SuperMix kit (Vazyme, China) according to the manufacturer's instructions.

Quantitative real-time PCR (qRT-PCR) was performed on a CFX96 Touch™ Real-Time PCR System (Bio-Rad, United States) using Taq Pro Universal SYBR qPCR Master Mix (Vazyme, China). A melting curve analysis was performed to confirm amplification specificity. The alfalfa *Actin* gene was used as an internal control for normalization, and relative expression levels were calculated using the 2^−ΔΔCt^ method.

### Drought treatment

Uniformly grown alfalfa seedlings propagated by cuttings at the same stage were transplanted into individual pots (8.5 × 8.5 × 9.5 cm) containing pre-weighed standard growth substrate (150 ± 5 g; vermiculite: nutrient soil = 2:1). All pots were placed in one tray filled with approximately 2 L of water and soaked overnight to allow complete saturation of the substrate. The following day, excess water was removed from the tray, and the pots were transferred to the greenhouse. Drought stress was imposed by withholding irrigation. To minimize microenvironmental variation and positional effects, pots were randomly rotated every 2 to 3 d throughout the experiment. Plant morphological changes were monitored regularly, and stress severity was assessed by observing progressive phenotypic symptoms such as leaf wilting. Key phenotypic parameters were recorded at the end of the stress period.

### Detection of the E3 ubiquitin ligase activity of MsPUB6

Based on bioinformatics analysis, the core U-box domain in MsPUB6 was mapped to amino acids 281 to 333 (Figure S1). The full-length MsPUB6 protein has a relatively large molecular mass (84.91 kDa) and tends to form inclusion bodies in the *Escherichia coli* prokaryotic expression system, resulting in extremely low soluble expression. To circumvent the difficulty in expressing the full-length protein while retaining the intact E3 catalytic core, we amplified a fragment spanning amino acids 251 to 550, which completely covers the U-box domain (281 to 333 aa). This fragment was inserted into the pGEX-4T-1 vector to generate an N-terminal GST-tagged fusion expression construct, designated pGEX-4T-1-MsPUB6 (251 to 550), for use in in vitro E3 ubiquitin ligase activity assays.

The recombinant plasmid pGEX-4T-1-MsPUB6 (251 to 550) and the empty pGEX-4T-1 vector were separately transformed into *E. coli* ArcticExpress competent cells. Positive single colonies were picked and cultured in liquid LB medium with shaking. When the OD_600_ reached 0.6 to 0.8, isopropyl β-D-thiogalactopyranoside (IPTG) was added to a final concentration of 0.2 mmol/L and induction was carried out overnight at 15 °C. Cells were harvested and disrupted by ultrasonication, and the soluble proteins in the supernatant were purified using GST affinity chromatography. The eluted protein fractions were dialyzed against phosphate-buffered saline overnight. SDS–PAGE and Western blot analysis confirmed that the purified protein migrated at the expected molecular weight, indicating that the desired protein had been successfully obtained for subsequent in vitro ubiquitination assays.

The in vitro ubiquitination reaction mixture consisted of E1 ubiquitin-activating enzyme, E2 ubiquitin-conjugating enzyme, Ub, purified GST–MsPUB6 fusion protein, and GST protein as a control. All reaction groups, including the negative and omission controls, contained reaction buffer and ATP. After mixing, the reaction was incubated at 37 °C for 4 h, terminated by boiling in protein loading buffer, and the samples were then subjected to Western blot analysis.

### Generation of *MsPUB6*-overexpression lines in alfalfa

The full-length coding sequence of *MsPUB6* was amplified from alfalfa cDNA. After sequence verification, the fragment was inserted into the *Sma*Ⅰ-digested Super1300 vector (Li et al. 2001) via homologous recombination. The resulting construct was then introduced into the *Agrobacterium tumefaciens* strain EHA105 for subsequent genetic transformation. Alfalfa transformation was performed following the method described by Fu's protocol (Fu et al. 2015), which involved callus induction, shoot differentiation, and root regeneration to obtain transgenic plants. To identify positive transformants, genomic DNA was first extracted from regenerated plants and subjected to PCR analysis using primers listed in Table S2. Subsequently, total RNA was extracted, and the expression level of *MsPUB6* was determined by qRT-PCR to confirm the establishment of overexpression lines.

### Virus-induced gene silencing (VIGS) in alfalfa

The *MsPUB6* coding sequence was analyzed using the SGN-VIGS online tool to identify a specific target fragment, and corresponding primers were designed and synthesized. The target fragment was cloned into the pTRV2 viral silencing vector, and the resulting construct was confirmed by sequencing prior to the transformation into the *A. tumefaciens* strain EHA105. Alfalfa leaves were infiltrated with the *Agrobacterium* cultures following the method described by Liu et al. (2023) to generate *MsPUB6*-silenced plants. Plants transformed with the empty pTRV2 vector were used as negative controls. After infiltration, plants were maintained under appropriate growth conditions. At the appropriate developmental stage, total RNA was extracted, and the expression level of *MsPUB6* was assessed by qRT-PCR. Lines with significant silencing efficiency were selected for subsequent drought tolerance assays.

### Generation of CRISPR-Cas9-edited mutant

Target sites within the gene of interest were first designed using the Alfalfa Genome sgRNA Design Platform (http://alfalfagedb.liu-lab.com/target/sgRNA_target/). Two expression cassettes, each containing a single gRNA, were then assembled into the CRISPR 2.0 vector (Zhao et al. 2024). The verified recombinant plasmids were subsequently introduced into the *A. tumefaciens* strain EHA105, and alfalfa genetic transformation was carried out following the method described by Fu et al. (2015). Regenerated plants were initially screened by PCR for the presence of the hygromycin resistance gene using primers listed in Table S2. For PCR-positive plants, genomic DNA was further amplified with Cas9-specific primers (Table S2). After purification, the amplified products were either cloned into the pCE2 TA/Blunt-Zero vector (Vazyme, Nanjing, China) for TA cloning and Sanger sequencing, or subjected to Hi-TOM high-throughput sequencing with a depth of > 5,000 reads. Mutation types were finally analyzed using SnapGene software.

### Measurement of relative leaf water content (RWC)

Approximately 0.1 g of fresh leaf tissue was collected and immediately weighed, and the weight was recorded as fresh weight (FW). The samples were then submerged in 10 mL of deionized water in 15 mL centrifuge tubes and allowed to soak for 6 h in the dark at 25 °C to achieve full hydration. After the soaking period, the samples were gently blotted dry with filter paper to remove excess surface water, and a second weight was recorded as turgid weight (TW). The samples were subsequently dried in a forced-air oven at 65 °C for 72 h, after which a third weight was recorded as dry weight (DW). Relative water content (RWC) was calculated using the following formula: RWC (%) = (FW – DW)/(TW – DW) × 100.

### Electric conductivity measurement

Approximately 0.1 g of fresh leaf tissue was collected, cut in half from the middle, and placed in a 15 mL polypropylene centrifuge tube containing 14 mL of deionized water. The tubes containing the samples were equilibrated on an orbital shaker at 70 rpm for 24 h at 25 °C, after which the initial conductivity (R1) was measured. Sterile deionized water was used as a blank control to determine the background conductivity (R0). The samples were then boiled at 100 °C for 30 min. After cooling to room temperature, the maximum conductivity (R2) and the corresponding blank control (R0') were measured. Electrolyte leakage (relative conductivity) was calculated using the following formula: Conductivity (%) = (R1 − R0)/(R2 − R0') × 100.

### Determination of chlorophyll content

Chlorophyll was extracted using the ethanol immersion method. Approximately 0.1 g of fresh leaf tissue was placed in a 15 mL centrifuge tube containing 10 mL of 95% ethanol and incubated in the dark for 48 h until complete decolorization. The absorbance of the extract was measured at 665 and 649 nm using a spectrophotometer (Thermo Fisher Scientific, United States). Chlorophyll contents were calculated according to the following formulas:


$$\mathrm{Chlorophyll}\;a\;(\mathrm{mg}/g\;\mathrm{FW})=(13.95\;\times \;A_{665}\;\text{–}\;6.88\;\times \;A_{649})\;\times \;0.01÷0.1$$

$$\mathrm{Chlorophyll}\;b\;(\mathrm{mg}/g\;\mathrm{FW})=(24.96\;\times \;A_{649}\;\text{–}\;7.32\;\times \;A_{665})\;\times \;0.01÷0.1$$

Totalchlorophyll(mg/gFW)=Chlorophylla+Chlorophyllb

### NBT and DAB staining

Fresh leaves of similar positions were collected from different alfalfa lines after treatment and used for nitroblue tetrazolium (NBT) and 3,3'-diaminobenzidine (DAB) staining to detect superoxide anion (O_2_^-^) and hydrogen peroxide (H_2_O_2_) accumulation, respectively. For NBT staining, leaves were fully immersed in 0.2% (w/v) NBT solution prepared in 50 mM sodium phosphate buffer (pH 7.8) and incubated in the dark at room temperature (approximately 25 °C) for 8 to 12 h (overnight). For DAB staining, leaves were soaked in staining solution containing 1 mg/mL DAB (adjusted to pH 3.8 with HCl) and Na_2_HPO_4_, and incubated under the same conditions. After incubation, leaves were thoroughly rinsed with distilled water to remove excess staining solution, then transferred to centrifuge tubes containing absolute ethanol and decolorized in a boiling water bath (95 to 100 °C) for 30 min. Decolorized leaves were subsequently immersed in 60% glycerol for photography.

### Determination of malondialdehyde (MDA), hydrogen peroxide (H_2_O_2_) contents, superoxide dismutase (SOD), and catalase (CAT) activities

Physiological indices, including MDA content, H_2_O_2_ content, SOD activity, and CAT activity, were quantified using commercial assay kits (Nanjing Jiancheng Bioengineering Institute, China). All measurements were performed strictly in accordance with the manufacturer's instructions.

### Electrophoretic mobility shift assay (EMSA)

EMSA was performed using the LightShift Chemiluminescent EMSA Kit (Thermo Scientific) according to the manufacturer's instructions. A probe containing the GCCGAC *cis*-element from the *MsPUB6* promoter was designed, synthesized, and labeled with biotin. Purified His-MsDREB1C recombinant protein was incubated with the biotin-labeled DNA probe to assess binding activity. For competition assays, a 50- or 100-fold excess of unlabeled competitor probes was added to the reaction mixture together with the biotin-labeled probe and His-MsDREB1C protein. To verify binding specificity, His-MsDREB1C protein was also incubated with a mutant probe carrying a mutated *cis*-element. Following the binding reactions, protein-DNA complexes were resolved on a nondenaturing polyacrylamide gel, transferred to a nylon membrane, and detected using a highly sensitive chemiluminescence imaging system (ChemiScope 6100, Shanghai, China).

### Luminescence imaging and dual-luciferase reporter assay

The promoter region of *MsPUB6* was cloned and inserted into the pGreenII 0800-LUC vector via homologous recombination to generate the reporter construct. Meanwhile, the coding sequence of MsDREB1C was inserted into the Super1300 vector to generate the effector construct. After sequence verification, both constructs were transformed into the *A. tumefaciens* strain GV3101. Agrobacterium suspensions carrying the effector (Super1300-MsDREB1C or empty vector) and the reporter were mixed at equal volumes and incubated at room temperature in the dark for 2 h. The mixed suspensions were then infiltrated into tobacco (*Nicotiana benthamiana*) leaves using a needleless syringe, and the infiltrated areas were marked. The plants were grown for an additional 48 h before leaf samples were collected for analysis.

For luminescence imaging, detached tobacco leaves were evenly sprayed with D-luciferin potassium salt working solution (150 mg/mL) until the leaf surface was fully moistened but not dripping, followed by dark adaptation for 5 min. Luminescence signals were then captured using a low-light cooled CCD camera system with an exposure time of 1 to 2 min. For dual-luciferase quantitative assays, leaf discs were collected from the infiltrated areas using a hole punch and immediately frozen in liquid nitrogen. Firefly luciferase (LUC) and Renilla luciferase (REN) activities were measured using the Dual-Luciferase Reporter Assay System (Promega, United States) following the manufacturer's instructions. Promoter activity was expressed as the LUC/REN ratio.

### Statistical analysis

Raw data were organized using Microsoft Excel and then subjected to statistical analysis with SPSS 22.0 software (IBM, United States). Differences were assessed by one-way ANOVA followed by Duncan's multiple range test for post hoc comparisons. A significance threshold of *P* < 0.05 was used to indicate statistically significant differences.

## Supplementary Material

kiag679_Supplementary_Data

## Data Availability

The authors confirm that all experimental data are available and accessible via the main text and/or the Supplementary data.
