# Supplementary material for: A GWAS-identified U-box E3 ligase MsPUB6 is positively regulated by MsDREB1C and contributes to drought tolerance in alfalfa
Source: Plant Physiol. 2026 Sep 11;202(2):kiag679. doi: 10.1093/plphys/kiag679 (PMC13650487; doi:10.1093/plphys/kiag679)
Supplement: Supplementary file 1 [file kiag679_supplementary_data.zip › 0 MsPUB-revised Supplementary materials.docx]

**A GWAS-identified U-box E3 ligase MsPUB6 is positively regulated by MsDREB1C and contributes to drought tolerance in alfalfa**

Yangyang Zhang,^1,†^ Xueqian Jiang,^1,†^ Huajuan Leng,^1^ Tian Zhang,^1^ Jing Cui,^1^ Ying Dong,^1^ Qingchuan Yang,^1,*^ Tiejun Zhang, ^1,*^ Junmei Kang^1,*^

^1^Institute of Animal Science, Chinese Academy of Agricultural Sciences, Beijing, 100193, China

^*^ Author for correspondence: [kangjunmei@caas.cn](mailto:kangjunmei@caas.cn) (Junmei Kang), [zhangtiejun@caas.cn](mailto:zhangtiejun@caas.cn) (Tiejun Zhang), [yangqingchuan@caas.cn](mailto:yangqingchuan@caas.cn) (Qingchuan Yang)

^†^These authors contributed equally to this work.

The author responsible for distribution of materials integral to the findings presented in this article in accordance with the policy described in the Instructions for Authors (https://academic.oup.com/plphys/pages/General-Instructions) is: Junmei Kang ([kangjunmei@caas.cn](mailto:kangjunmei@caas.cn)).

**Running head: MsDREB1C-MsPUB6 regulates alfalfa drought tolerance**

**Supplementary Figure S1** Multiple sequence alignment of MsPUB6 with orthologs from other species. The alignment includes sequences from *Medicago truncatula* (XP_003601808.1), *Glycine max* (KAH1226877.1), *Trifolium repens* (KAK2384100.1), *Arabidopsis thaliana* (NP_174112.1), and *Zea mays* (NP_001169575.1).

**Supplementary Figure S2** Tissue-specific expression analysis of *MsPUB6*. Error bars represent the mean ± standard error (SEM) of three biological replicates. Statistical significance among groups was assessed by one-way ANOVA, and different lowercase letters indicate significant differences (*P* < 0.05) based on Duncan’s multiple range test.

**Supplementary Figure S3** qRT-PCR analysis of *MsPUB6* expression under cold (4 °C), salt (200 mM NaCl) and ABA (100 μM) treatment, respectively. Error bars represent the mean ± standard error (SEM) of three biological replicates. Statistical significance among groups was assessed by one-way ANOVA, and different lowercase letters indicate significant differences (*P* < 0.05) based on Duncan’s multiple range test.

**Supplementary Figure S4** Validation of overexpression lines and measurement of physiological indices. **a)** Relative expression levels of *MsPUB6* in *MsPUB6*-overexpressing lines (OE19 and OE20) compared with wild-type. **b)** Malondialdehyde (MDA) content in leaves of wild-type (WT) and *MsPUB6*-overexpressing (*MsPUB6*-OE) lines after drought treatment. **c)** Hydrogen peroxide (H₂O₂) content in leaves of WT and *MsPU6B*-OE lines after drought treatment. **d)** Catalase (CAT) activity in leaves of WT and *MsPUB6*-OE lines after drought treatment. Error bars represent the mean ± standard error (SEM) of three biological replicates. Statistical significance among groups was assessed by one-way ANOVA, and different lowercase letters indicate significant differences (*P* < 0.05) based on Duncan’s multiple range test.

**Supplementary Figure S5** Sanger sequencing of single colonies confirmed CRISPR-Cas9-induced mutations at target site 1 and 2 in the mutant lines *msdreb1c-25* and *msdreb1c-26*.

**Supplementary Figure S6** Comparison of physiological indices between CRISPR-Cas9-mediated *msdreb1c* mutants and wild-type after drought stress. **a)** Malondialdehyde (MDA) content. **b)** Hydrogen peroxide (H₂O₂) content. **c)** Superoxide dismutase (SOD) activity. **d)** Catalase (CAT) activity. Error bars represent the mean ± standard error (SEM) of three biological replicates. Statistical significance among groups was assessed by one-way ANOVA, and different lowercase letters indicate significant differences (*P* < 0.05) based on Duncan’s multiple range test.

**Supplementary Table S1.** Analysis of promoter elements of *MsPUB6.*

| *Cis*-acting element | Number | Function |
| --- | --- | --- |
| ABRE | 5 | *cis*-acting element involved in the abscisic acid responsiveness |
| G-Box | 4 | *cis*-acting regulatory element involved in light responsiveness |
| ERE | 2 |  |
| CGTCA-motif | 1 | *cis*-acting regulatory element involved in the MeJA-responsiveness |
| TGACG-motif | 1 | *cis*-acting regulatory element involved in the MeJA-responsiveness |
| MYB | 3 |  |
| Myb | 3 |  |
| MYC | 5 |  |
| Box 4 | 8 | part of a conserved DNA module involved in light responsiveness |
| **DRE core** | **2** |  |
| TGA-element | 2 | auxin-responsive element |
| MRE | 1 | MYB binding site involved in light responsiveness |
| I-box | 1 | part of a light responsive element |
| LAMP-element | 1 | part of a light responsive element |
| MBS | 2 | MYB binding site involved in drought-inducibility |
| CAT-box | 1 | *cis*-acting regulatory element related to meristem expression |
| ARE | 2 | *cis*-acting regulatory element essential for the anaerobic induction |

**Supplementary Table S2.** List of primer pairs and used in this study.

| **Primer** | **Sequence (5'-3')** |
| --- | --- |
| **Primers amplified for MsDREB1C-mutant constructs** | |
| MsDREB1C-2.0-F1 | TAGGTCTCAATCGAACAAAGCACCAGTG |
| MsDREB1C-2.0-R1 | GCGGTCTCAGCCACCCGGTATTGCACCAGCCGGGAA |
| MsDREB1C-2.0-F2 | TAGGTCTCATGGCGAGTCTCCGTTTTAGAGCTAGAAA |
| MsDREB1C-2.0-R2 | GCGGTCTCACTTAGTCTTCTTTGCACCAGCCGGGAA |
| MsDREB1C-2.0-F3 | TAGGTCTCATAAGATTTGGCTGTTTTAGAGCTAGAA |
| MsDREB1C-2.0-R3 | TAGGTCTCAATATAAAAAAAGCACCGACTCGGTGCC |
| JC-2.0-F | catcgaccggaagaggtaca |
| JC-2.0-R | acgccagctggcgaaagggg |
| **Primers amplified for detecting gene editing plants** | |
| Hygromycin-F | ATCCTGCAAGCTCCGGATGCCTC |
| Hygromycin-R | CGTCTCCGACCTGATGCAGCTCT |
| MsDREB1C-Cas9-JC-F | GTGAGGTTAGCTGCGACGA |
| MsDREB1C-Cas9-JC-R | TGCAAAGTTGAGACAGGCG |
| **Other primers** | |
| MsPUB6-RT-F | CCCTGTTCTCCTAACGGTGT |
| MsPUB6-RT-R | TTCTGGTGGAAGGGGCATTT |
| MsACTIN-RT-F | CAAAAGATGGCAGATGCTGAGGAT |
| MsACTIN-RT-R | CATGACACCAGTATGACGAGGTCG |
| MsPUB6-F | ATGATGGATGTTTCTGAGGTTGAAGAA |
| MsPUB6-R | CTAACACTGGTAAACAGAATAGCTTTTG |
| MsPUB6-1300-F | tctagaaagcttctgcaggggATGATGGATGTTTCTGAGGTTGAAGAA |
| MsPUB6-1300-R | ctcctcgcccttgctcaccatACACTGGTAAACAGAATAGCTTTTG |
| 1300-F | ACAACGCTTTACAGCAAGAACGG |
| 1300-R | GGTCAGGGTGGTCACGAGGG |
| MsDREB1C-1300-F | tctagaaagcttctgcaggggATGGATATGTTAACAACTAACAACTC |
| MsDREB1C-1300-R | ctcctcgcccttgctcaccatATAATTCCATAGTGATACCTCTTC |
| MsPUB6-TRV2-F | ctctagaaggcctccatggg GGAGCTATGG CTTTGTTCAA |
| MsPUB6-TRV2-R | cgtgagctcggtaccggatc GAGGTTCGAA ATATTTGAGG |
| TRV2-F | CATTGTTACTCAAGGAAGCA |
| Nos-R | GATAATCATCGCAAGACCGG |
| MsDREB1C-SP6-F | TTCAGAGCGATAACGCGATGGATATGTTAACAACTAACAACTC |
| MsDREB1C-SP6-R | CGAGCCCGAATTCGTTTTTAATAATTCCATAGTGATACCTCTTC |
| proMsPUB6-LUC-F | TGATATCGAATTCCTGCAGCCCTTTCCATTTT AAACTCCTTA AC |
| proMsPUB6-LUC-R | TGGCGGCCGCTCTAGAACTAGTATTAATAAAA TATATTAATT AAATTCATC |

**Supplementary Table S3.** List of probes used in this study

| **Probes** | **Sequence (5'-3')** |
| --- | --- |
| MsPUB6-F-biotin | ACAAACAAGGAGGTCGGCAACTGTCGGCCGGTGAAGCGGGG |
| MsPUB6-F | ACAAACAAGGAGGTCGGCAACTGTCGGCCGGTGAAGCGGGG |
| MsPUB6-R | CCCCGCTTCA CCGGCCGACA GTTGCCGACC TCCTTGTTTG T |
| MsPUB6-mutant-F-biotin | ACAAACAAGGAGtTttttAACTtTttttcggTGAAGCGGGG |
| MsPUB6-mutant-R | CCCCGCTTCACCGAAAAAAAGTTAAAAAACTCCTTGTTTGT |
